# Association between adverse childhood experiences and the number of suicide attempts in lifetime

**DOI:** 10.1192/j.eurpsy.2023.1182

**Published:** 2023-07-19

**Authors:** J. Andreo-Jover, E. Fernandez-Jimenez, J. Curto-Ramos, N. Angarita-Osorio, N. Roberto, A. De la Torre-Luque, A. Cebria, M. Diaz-Marsa, M. Ruiz-Veguillla, J. B. Bobes Garcia, M. Fe Bravo Ortiz, V. Perez Solá

**Affiliations:** 1 Hospital La Paz Institute for Health Research (IdiPAZ); 2 Department of Psychiatry, Universidad Autónoma de Madrid (UAM); 3 Instituto de Investigación del Hospital Universitario La Paz (IdiPAZ); 4 Department of Psychiatry, Clinical Psychology and Mental Health, La Paz University Hospital, Madrid; 5 Mental Health Research Group, Hospital del Mar Medical Research Institute (IMIM); 6 Bipolar and Depressive Disorders Unit, Hospital Clinic, Institute of Neurosciences, University of Barcelona, IDIBAPS, CIBERSAM, Barcelona; 7 Departamento de Medicina Legal, Psiquiatría y Patología, Centro de Investigación Biomédica en Red en Salud Mental (CIBERSAM), Madrid; 8 Hospital Parc-Taulí, Barcelona; 9 Hospital Clínico San Carlos, Madrid; 10 Hospital Virgen del Rocio, Sevilla; 11 Universidad de Oviedo-HUCA, Oviedo; 12 Hospital La Paz, Madrid, Spain

## Abstract

**Introduction:**

Adverse childhood experiences (ACEs), defined as abuse, neglect, or a dysfunctional household in childhood, have been associated with suicidality (Fjeldsted et al., 2020). Every type of ACE has a direct impact on suicide ideation, self-harm and/or suicide attempt (Angelakis et al., 2019).

**Objectives:**

We aim to quantify the association between types of ACEs (including emotional, physical, sexual abuse, and emotional and physical neglect) and the number of suicide attempts in lifetime.

**Methods:**

We included 748 patients who attempted suicide at least once. They were asked to complete the Columbia-Suicide Severity Rating Scale (CSSRS), and the Childhood Trauma Questionnaire-Short Form (CTQ-SF). Logistic regression models were run to assess the association between each ACE type and the number of suicide attempts.

**Results:**

Poisson univariate regression analyses show a linear trend in the relationship between having a higher number of suicide attempts and having suffered every ACE type in childhood (p<0.05). Our results show a lower percentage of previous suicide attempts among participants without ACEs, and an increasing tendency among patients with various types of ACEs. The rate of ACEs types is significantly higher in the group with previous suicide attempts than in the first-attempt group (p=0.000).

**Image:**

**Figure:**
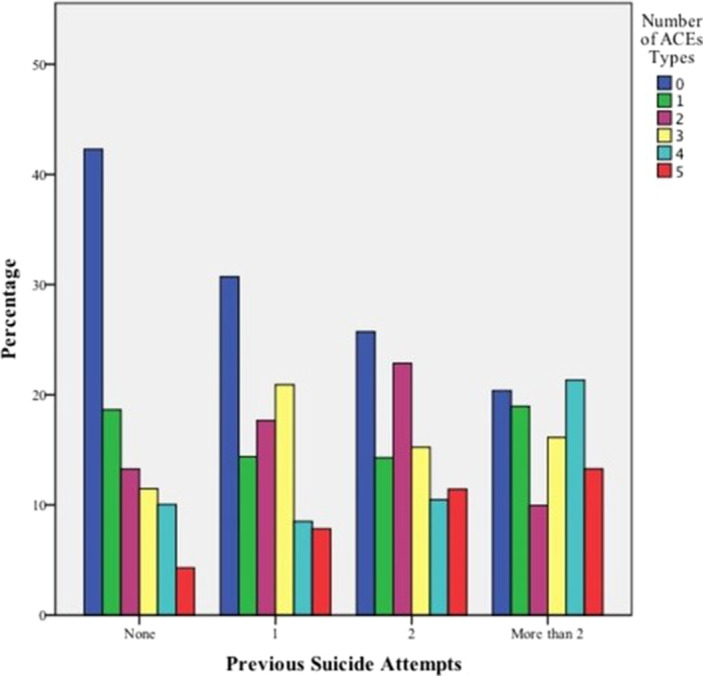

**Figure:**
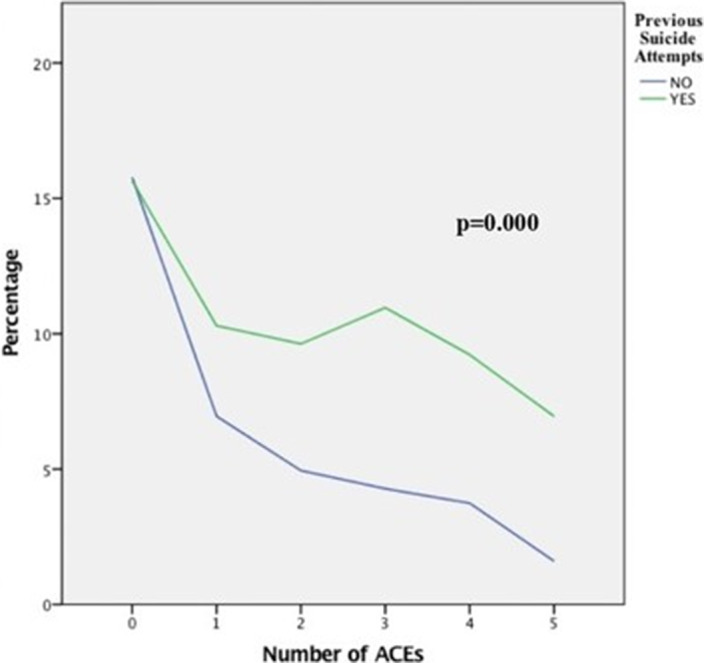

**Image 2:**

**Conclusions:**

This study contributes to clarify the role of childhood trauma in the number of suicide attempts in lifetime. This has important implications for reducing suicide rates, and preventing future re-attempts. Further studies analysing every construct of childhood trauma may contribute to the detection of suicidal behaviour.

**Fundings:**

This work was supported by the Instituto de Salud Carlos III (grant number: PI19/00941 SURVIVE) and co-funded by the European Union (grant numbers: COV20/00988, PI17/00768), the European Union’s Horizon 2020 research and innovation programme Societal Challenges (grant number: 101016127), and the Fundación Española de Psiquiatría y Salud Mental

**Acknowledgements:**

SURVIVE project (PI19/00941)

**Keywords:**

Suicide attempt, Adverse Childhood Experiences

**References:**

Angelakis, I., Gillespie, E. L., & Panagioti, M. (2019). Childhood maltreatment and adult suicidality: A comprehensive systematic review with meta-analysis. *Psychological Medicine*, 49(7), 1057-1078. https://doi.org/10.1017/S0033291718003823

Fjeldsted, R., Teasdale, T. W., & Bach, B. (2020). Childhood trauma, stressful life events, and suicidality in Danish psychiatric outpatients. *Nordic Journal of Psychiatry*, 74(4), 280-286. https://doi.org/10.1080/08039488.2019.1702096

**Disclosure of Interest:**

None Declared

